# Phenyl Ring: A Steric Hindrance or a Source of Different Hydrogen Bonding Patterns in Self-Organizing Systems?

**DOI:** 10.1021/acs.jpclett.1c00186

**Published:** 2021-02-24

**Authors:** Andrzej Nowok, Mateusz Dulski, Joanna Grelska, Anna Z. Szeremeta, Karolina Jurkiewicz, Katarzyna Grzybowska, Małgorzata Musiał, Sebastian Pawlus

**Affiliations:** † Institute of Physics, University of Silesia in Katowice, 75 Pułku Piechoty 1, 41-500 Chorzów, Poland; ‡ Silesian Center for Education and Interdisciplinary Research, 75 Pułku Piechoty 1A, 41-500 Chorzów, Poland; § Institute of Materials Engineering, University of Silesia in Katowice, 75 Pułku Piechoty 1A, 41-500 Chorzów, Poland

## Abstract

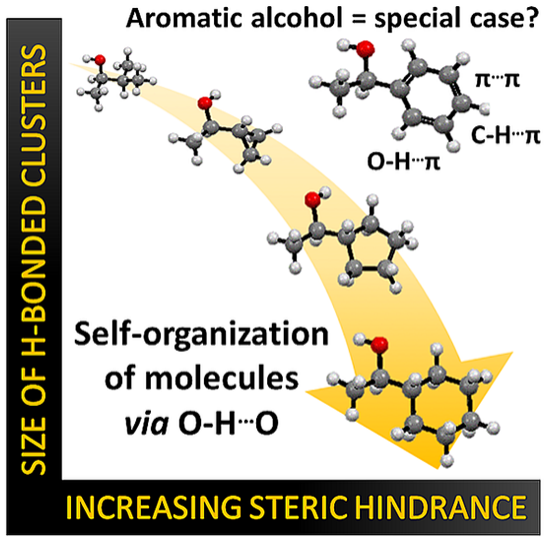

A series of five alcohols (3-methyl-2-butanol, 1-cyclopropylethanol, 1-cyclopentylethanol, 1-cyclohexylethanol, and 1-phenylethanol) was used to study the impact of the size of steric hindrance and its aromaticity on self-assembling phenomena in the liquid phase. In this Letter, we have explicitly shown that the phenyl ring exerts a much stronger effect on the self-organization of molecules via the O–H···O scheme than any other type of steric hindrance, leading to a significant decline in the size and concentration of the H-bonded clusters. Given the combination of calorimetric, dielectric, infrared, and diffraction studies, this phenomenon was ascribed to its additional proton-acceptor function for the competitive intermolecular O–H···π interactions. The consequence of this is a different packing of molecules on the short- and medium-range scale.

Hydrogen bonds are one of the most prevalent chemical interactions. Despite their relative weakness (0.2–40 kJ/mol), these interactions are a driving force for self-assembling phenomena in liquids, such as alcohols, amines, amides, and peptides.^1−3^ Consequently, glass-forming monohydroxy alcohols have become model self-organizing systems to dissect the relationship between the internal molecular architecture and the spatial arrangement of molecules in the liquid phase.^3−5^

In general, globular-shaped isomers with a hydroxyl group highly shielded by alkyl substituents tend to agglomerate in dimers, trimers, or higher-membered rings.^6−9^ In turn, terminal alcohols and their less-hindered analogues, like 6-methyl-3-heptanol, form supramolecular clusters with a rather chain-like architecture of H-bonds.^10−12^ This type of organization leads to the appearance of a characteristic large Debye process in the dielectric spectra, which reflects the mobility of the supramolecular structures with a nonzero resultant dipole moment.^3^ There is also a strong connection between the size of the supramolecular structures and the interference of neighboring substituents.^13−15^ Introduction of a steric hindrance, particularly in the vicinity of the hydroxyl group, reduces the surface area available for H-bond formation; weakens the H-bonds; and, as a consequence, attenuates the propensity for self-association.^13,14^ However, the magnitude of the exerted effect is dependent on the size and the number of both the neighboring and more distant groups.^12^ The situation becomes more complicated in phenyl alcohols, in which the bulky aromatic ring is a source of additional π···π, C–H···π, and intra- or intermolecular O–H···π interactions.^16−18^ For decades, the phenyl ring was regarded as a moiety able to prevent the self-organization.^19^ However, most recent studies proved that most phenyl alcohols, such as 1-phenyl-1-propanol or 4-phenyl-2-butanol, tend to agglomerate in H-bonded networks, just like their alkyl analogues (1-propanol and 2-butanol).^20,21^ Nevertheless, the Debye relaxation has never been detected as a separate peak in the dielectric spectra of aromatic monohydroxy alcohols, in contrast to their alkyl analogues.^19−23^ Various explanations have been proposed for this phenomenon, such as the balance between chain- and ring-like suprastructures or their small size due to the presence of a bulky steric hindrance.^16,20^ Despite numerous studies on this issue, the impact of the steric hindrance on the structural and dielectric properties has not been well understood yet because the Debye-like process remains visible in the case of even more sterically hindered *trans*-2-methylcyclohexanol.^7^

Considering the ongoing discussion, the question naturally arises: does the phenyl ring play only the role of a steric hindrance, or is it also a source of a different H-bonding pattern in the self-organizing systems? To answer this intriguing question, we decided to investigate five alcohols, namely, 3-methyl-2-butanol (3M2B), 1-cyclopropylethanol (1CPr1E), 1-cyclopentylethanol (1CPe1E), 1-cyclohexylethanol (1CH1E), and aromatic 1-phenylethanol (1P1E), in which the steric hindrance size increases from a small isopropyl or cyclopropyl group up to the six-membered cyclohexyl or phenyl ring (Figure 1a). Considering the combination of broadband dielectric (BDS) and Fourier transform infrared (FTIR) spectroscopy with calorimetric (DSC) and X-ray diffraction (XRD) techniques, we discuss to what extent the increasing steric hindrance affects the self-association process and what the role of the π-electron cloud is.

**Figure 1:**
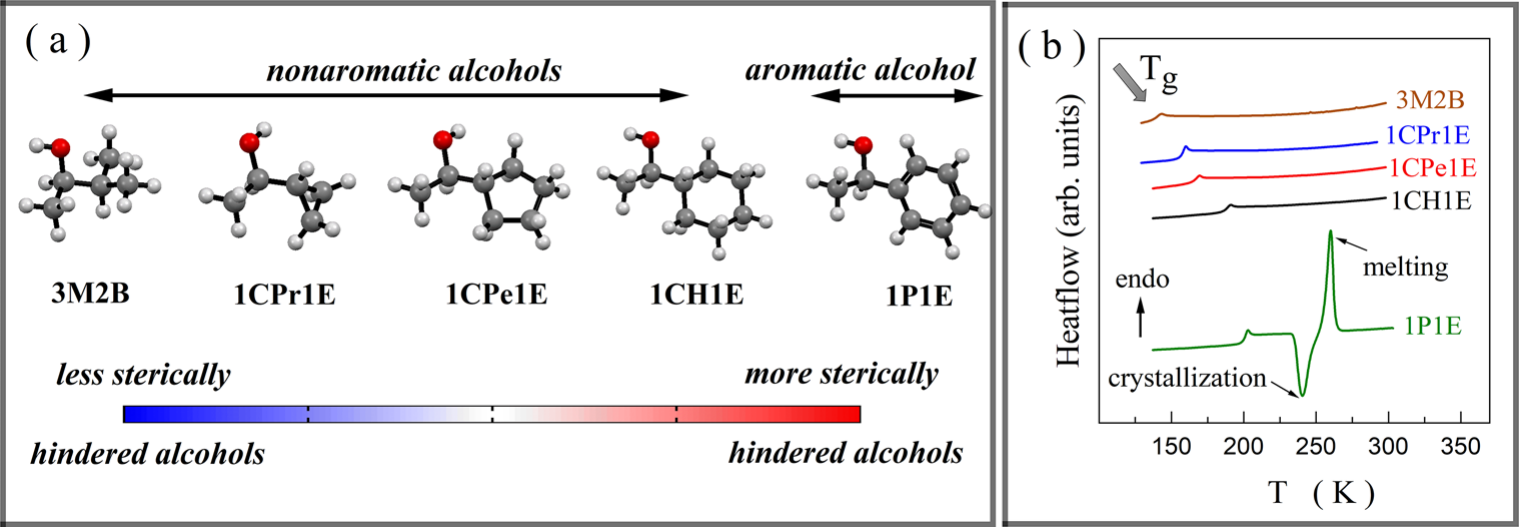
Overview of the molecular structure models for the studied alcohols (a). DSC thermograms collected while heating with a rate of 10 K min^–1^ (b).

Calorimetric studies show that all studied alcohols could be vitrified while cooling. In the series of alcohols containing the nonaromatic cyclic substituent, the glass transition temperature (*T*_g_) increases with their molar mass (Table 1). Such a tendency is in line with a general rule stating that *T*_g_ correlates with the molar mass of a molecule, *M*: *T*_g_(*M*) ∝ *M*^α^, with α being close to 0.5.^24^ However, this empirical law is violated when comparing *T*_g_ of 3M2B with 1CPr1E, and 1CH1E with 1P1E. Moreover, only the latter alcohol undergoes the crystallization process while heating with a rate of 10 K min^–1^ (Figure 1b). To explain these peculiarities, further dielectric studies were performed.

**Table 1:** Molar Mass (*M*), Glass Transition Temperature (*T*_g_), and Full Width at Half Maximum of the γ/δ OH Band (FWHM) at 298 K and *T*_g_ of the Studied Alcohols

compound	*M* (g mol^–1^)	*T*_g_ (K)	FWHM at 293 K (cm^–1^)	FWHM at *T*_g_ (cm^–1^)
3M2B	88	137(1)	228(4)	208(4)
1CPr1E	86	156(1)	237(4)	212(4)
1CPe1E	114	164(1)	233(4)	210(4)
1CH1E	128	186(1)	225(4)	208(4)
1P1E	122	198(1)	241(4)	222(4)

The dielectric loss spectra of the selected 1CH1E are shown in Figure 2a. A single relaxation is visible below *T*_g_ (186(1) K) within the frequency range of 10^–1^–10^6^ Hz. Such a feature along with low intensity and the symmetrical, broad shape of the relaxation peaks allows us to ascribe this process to the group of secondary relaxations. A similar image was obtained for the other alcohols except for 1P1E, for which no relaxation process can be detected below *T*_g_ (see the Supporting Information). Above *T*_g_, another relaxation process appears for all alcohols, characterized by well-separated maxima and much higher magnitude than the secondary mode (compare Figure 2a and Supporting Information). Because the peaks are narrow in each case, they can be treated as a combination of the dominating Debye process (connected with the mobility within the H-bonded structures) and structural α-relaxation (stemming from cooperative motions of molecules in liquid state). This hypothesis is supported by a good adjustment of the Debye function to the shape of the loss peak in 3M2B (see inset in Figure 2b) and previous studies on 1P1E or structurally related butanols.^25,26^ Consequently, because of the overlapping Debye and α modes, the identification of the secondary relaxation origin cannot be done. Nevertheless, in structurally related alcohols, *e.g.*, 1-propanol and 5-methyl-2-hexanol, the secondary process was successfully identified as the JG β-relaxation.^27^

**Figure 2:**
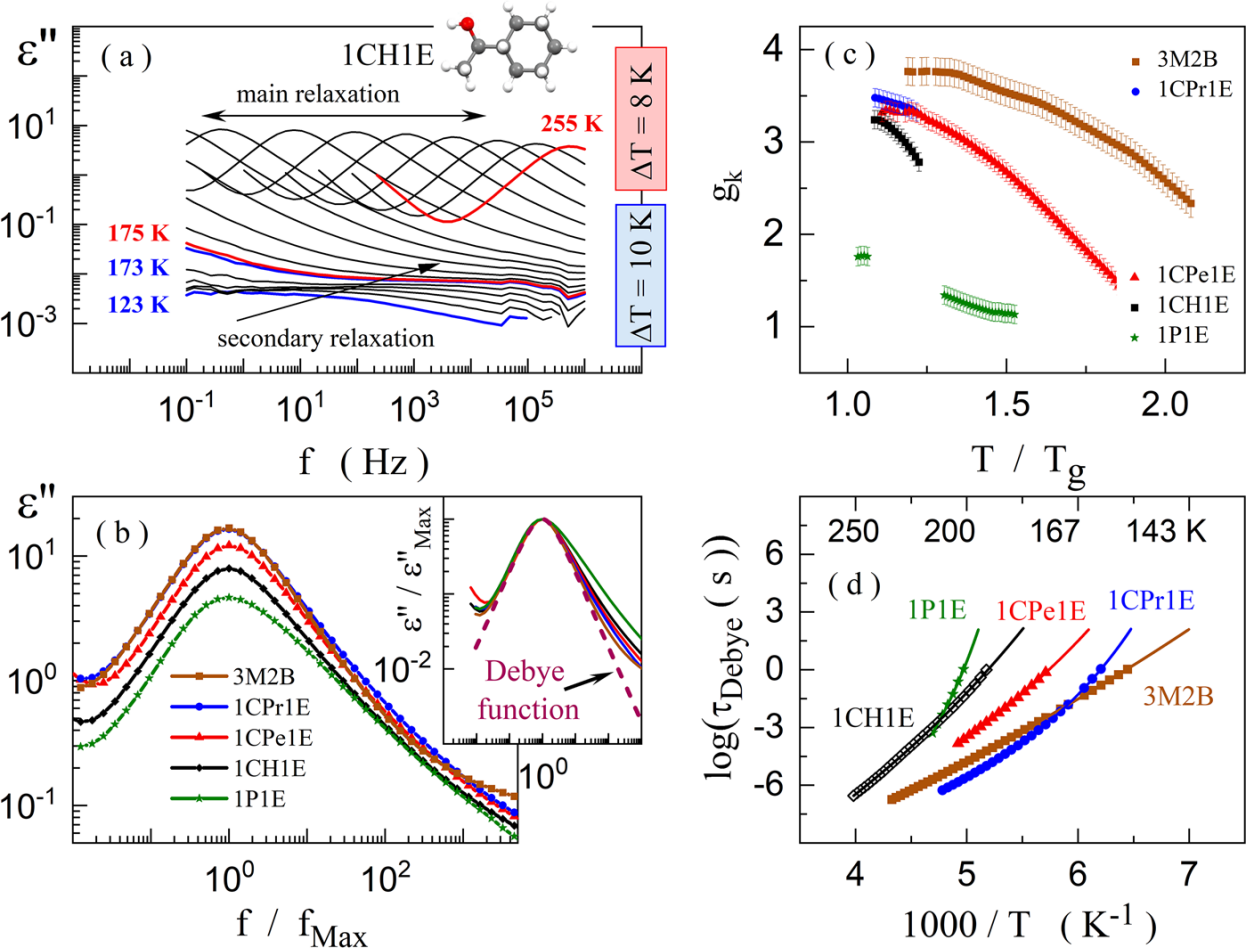
Dielectric loss spectra of 1CH1E (a). Comparison of the main relaxation process for the analyzed alcohols in terms of their amplitude and shape (b). Inset shows a comparison of the main process shape with the Debye function. Thermal evolution of the Kirkwood–Fröhlig factor (c) and the Debye relaxation time (d).

With increasing the steric hindrance size, the amplitude of the main relaxation peak declines (Figure 2b). Simultaneously, its high-frequency slope becomes asymmetrically broadened for 1CPr1E, 1CPe1E, 1CH1E, and 1P1E. Such a scenario is possible when H-bonded supramolecular structures become smaller.^3,28^ The broadening, although relatively small for all alcohols with the nonaromatic cyclic substituent, increases with the size of the steric hindrance. Hence, the dielectric measurements suggest that the size of the H-bonded clusters declines with increasing steric hindrance. Interestingly, the shape of the main relaxation peak is fundamentally different in the case of 1P1E (see green line in the inset of Figure 2b). To explain this discrepancy, the temperature evolution of the Kirkwood–Fröhlich factor, *g*_k_, was analyzed.

This parameter provides information on the cross-correlation of dipole moments between adjacent molecules and is defined by the formula *g*_k_ = , where *k*_B_ is Boltzmann’s constant, *M* molar mass, ε_0_ vacuum permittivity, ε_s_ static dielectric permittivity, ε_∞_ dielectric permittivity at infinite frequencies, *N*_A_ Avogadro number, ρ density, and μ molecular dipole moment.^29^ According to the Dannhauser model, positive (*g*_k_ > 1) and negative (*g*_k_ < 1) cross-correlation between neighboring molecules in self-assembling systems indicates their chain- and ring-like organization within the aggregates, respectively.^6,30,31^ For calculations, the ε_s_ was taken from dielectric spectra, whereas the ρ and ε_∞_ values were estimated based on the temperature-dependent measurements of density and refractive index, *n* (ε_∞_ ≈ *n*^2^). As shown in Figure 2c, *g*_k_ is greater than 1 in the whole temperature range for all studied alcohols and increases when approaching *T*_g_. In the vicinity of *T*_g_, *g*_k_ varies only to a small extent among the nonaromatic alcohols, taking the highest values for 2M1B and the lowest for the most sterically hindered 1CH1E. In contrast, *g*_k_ is substantially smaller for 1P1E in the whole temperature range. Assuming the Dannhauser model, the thermal evolution of *g*_k_ indicates that the molecules of all studied alcohols tend to organize themselves into chain-like associates whose size and/or concentration increase while cooling. Nevertheless, the concentration and/or the morphology of clusters are different in 1P1E. This finding coincides with the substantial broadening of the main relaxation peak of 1P1E compared to the other nonaromatic alcohols.

The differences in the morphology of the H-bonded aggregates have also been reflected in their molecular dynamics. The Debye relaxation times, determined according to the procedure described in Supporting Information, increase while cooling in a nonlinear way for each alcohol, indicating that the mobility of the associates becomes slower when approaching *T*_g_ (Figure 2d). However, the effect on the relaxation dynamics exerted by the temperature change is much stronger for the stiffer alcohols (compare 1P1E with 1CH1E and 1CPr1E with 3M2B). Hence, the elasticity of the steric hindrance has a huge impact on the pace of the temperature-induced H-bonded chain restructuring.

FTIR spectra of the studied alcohols were analyzed in the 3050–3650 cm^–1^ range, in which the stretching modes of the OH groups appear. As shown in Figure 3a, the FTIR spectrum of the selected liquid 1CH1E collected at 293 K is characterized by a single broad OH stretching band centered at 3347 cm^–1^. A similar image was obtained for the other nonaromatic alcohols (see the Supporting Information). According to the literature, this intense band stems from the stretching modes of those OH moieties, which are only proton donors (γ OHs) or both proton donors and acceptors in H-bonded multimeric supramolecular structures (δ OHs).^3^ The position of the γ/δ-band maximum is similar at 293 K for all studied alcohols (Figure 3c). It means that the intermolecular H-bonds within the self-assemblies are of comparable strength for all alcohols, despite considerable differences in their steric hindrance size. In turn, a change in the substituent type to the aromatic phenyl ring modifies the spectral character leading to the appearance of a second, less-intense OH stretching band centered at 3553 cm^–1^ (Figure 3b). Interestingly, this band does not occur for the nonaromatic alcohols including 1CH1E, which has almost the same steric hindrance size as 1P1E. This band results from the OH groups, which are not involved in the association of molecules via the O–H···O scheme (α/β OHs).^3^ Furthermore, free hydroxyl moieties are identified in O–H···π intermolecular interactions in liquid aromatic alcohols.^16^ Therefore, 1P1E is characterized by a lower concentration of the H-bonded chain-like structures than the other studied alcohols. In turn, the degree of association, estimated based on the integrated intensity analysis, is similar for all nonaromatic alcohols, independent of the size of the steric hindrance.

**Figure 3:**
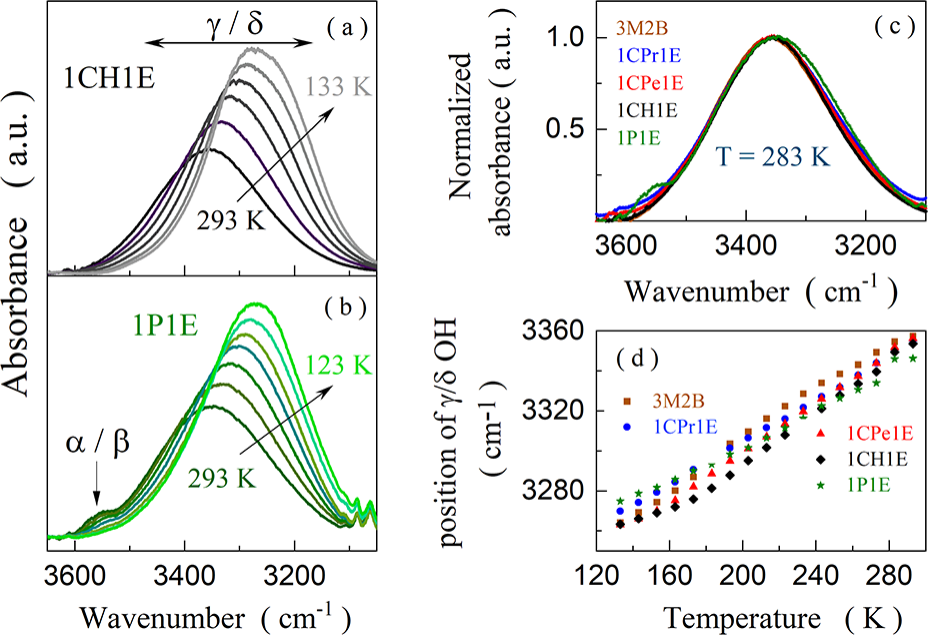
Infrared spectra of 1CH1E (a) and 1P1E (b) in the 3650–3050 cm^–1^ range, measured from 293 to 133 K. Comparison of the OH stretching bands for the studied alcohols at 283 K (c). Temperature dependences of the γ/δ OH stretching band position (d).

The γ/δ-band shifts toward lower wavenumbers (red-shift) while cooling (Figure 3d). The thermal evolution of the position of this band is similar for all systems, despite differences in their *T*_g_. The γ/δ-band becomes more intense and narrower when approaching *T*_g_. The latter effect is reflected in its full width at half-maximum (FWHM), which decreases for all alcohols while cooling (see Table 1). Additionally, the α/β-band of 1P1E becomes less intense when lowering the temperature, but it does not vanish even in the very vicinity of *T*_g_. All these features suggest a temperature-induced molecular rearrangement, which involves O–H···O bond strengthening, and an increase in the H-bonded agglomerates’ size, concentration, and homogeneity. Such a conception correlates with the rise in the *g*_k_ factor when approaching *T*_g_. In the case of 1P1E, the rearrangement of molecules reduces the population of free OH groups. Nonetheless, they still occur even below *T*_g_. Besides, 1P1E is characterized by the highest fwhm values of the γ/δ-band in the whole temperature range (Table 1). This means that despite the growing degree of the association while cooling, the aromatic alcohol remains the most disordered system among the studied substances in terms of the H-bonding pattern. The difference between nonaromatic alcohols and 1P1E becomes particularly distinguishable around *T*_g_, where the FWHM takes similar values for all alcohols except for 1P1E. Hence, introducing the π···π or OH···π interactions exerts a much stronger effect on the molecular self-organization than the change in the steric hindrance size. The former modification leads to a fundamentally different H-bond network morphology and intermolecular structure. The latter results in different sizes of the supramolecular associates and their concentration.

The differences in the association ability via H-bonds of the studied alcohols are reflected in the XRD data. The origin of two main diffraction peaks, visible in Figure 4a, has already been discussed in detail for various alcohols and other H-bonded liquids.^32−35^ The main scattering peak at about 1.3 Å^–1^ corresponds to an average correlation length between neighboring molecules. In turn, the prepeak, visible here at about 0.65 Å^–1^, originates from the correlations between OH groups in neighboring OH skeletons formed when molecules link through H-bonds into larger supramolecular aggregates. The prepeak is a signature of the medium-range order extending beyond the first shell of neighbors. 3M2B exhibits the highest amplitude and the smallest width of the prepeak. This is in line with the highest *g*_k_ values for this alcohol and signifies the highest degree of the medium-range order compared to other alcohols. As expected, the introduction of the steric hindrance in the form of any ring significantly reduces the prepeak’s intensity and increases its width because of inhibition of the self-association phenomena. The bigger the size of the cyclic substituent, the stronger the damping of the prepeak. Some residual prepeak is still present for 1P1E, but it is significantly suppressed compared to 1CH1E, despite their very similar molecular structure. Moreover, the huge difference between the intermolecular structure of these two alcohols is pronounced when one sets together their main diffraction peaks. The aromatic alcohol has a much more disordered short-range arrangement of molecules and a much wider distribution of the nearest-neighbor intermolecular distances than the 1CH1E. In other words, the nanoscale structure is much more inhomogeneous for 1P1E. Upon lowering the temperature, the diffraction peaks for each alcohol get sharper as the coherence lengths of the short- and medium-range order increase (see data for 1CH1E in Figure 4b and other alcohols in the Supporting Information). This behavior agrees with the conclusions derived from the Kirkwood–Fröhlich factor analysis, indicating the growth of the chain-like associates with the temperature drop.

**Figure 4:**
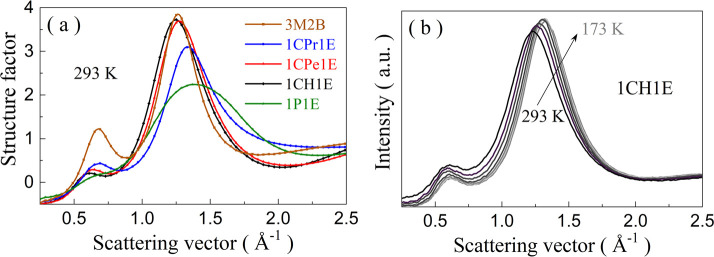
X-ray diffraction data in the form of the structure factors in the low scattering vector range of 0.25–2.5 Å^–1^ measured at 293 K (a). Temperature evolution of the diffraction patterns for 1CH1E from 293 K down to 173 K (b).

To sum up, the self-assembling processes in alcohols depend on both the size and the type of steric hindrance. The increase in the steric hindrance dimension leads to a decline in the size of H-bonded supramolecular clusters. In contrast, the phenyl ring plays a double role, acting as a steric hindrance and a source of a different (more heterogeneous) H-bonding pattern in self-organizing alcohols due to π···π and OH···π interactions. Consequently, the aromatic ring exerts a much stronger effect on the self-assembling phenomena, introducing disorder in hydrogen bond structure and affecting the supramolecular organization on the medium-range scale.

## Supporting Information Available

Experimental details, as well as additional analyses of dielectric, infrared, and diffraction data (PDF)
