# Supplementary material for: Phenyl Ring: A Steric Hindrance or a Source of Different Hydrogen Bonding Patterns in Self-Organizing Systems?
Source: J Phys Chem Lett. 2021 Feb 24;12(8):2142–7. doi: 10.1021/acs.jpclett.1c00186 (PMC8034769; doi:10.1021/acs.jpclett.1c00186)
Supplement: Supplementary file 1 — jz1c00186_si_001.pdf [file jz1c00186_si_001.pdf]

Phenyl Ring: A Steric Hindrance or a Source of Different Hydrogen Bonding Patterns in Self-Organizing Systems?

Supplementary materials

Andrzej Nowok[a,*], Mateusz Dulski[b,c], Joanna Grelska[a,b], Anna Z. Szeremeta[a], Karolina Jurkiewicz[a,b], Katarzyna Grzybowska[a,b], Małgorzata Musiał[a,b] and Sebastian Pawlus[a,b]

[a]*Institute of Physics, University of Silesia in Katowice, 75 Pułku Piechoty 1, 41-500 Chorzów, Poland*

[b]*Silesian Center for Education and Interdisciplinary Research, 75 Pułku Piechoty 1A, 41-500 Chorzów, Poland*

[c]*Institute of Materials Engineering, University of Silesia in Katowice, 75 Pułku Piechoty 1A, 41-500 Chorzów, Poland*

*email: andrzej.nowok@smcebi.edu.pl

1. Experimental details

1.1. Density measurements

The density, ρ, of investigated samples was determined using vibrating-tube densimeter DMA 4500 M (Anton Paar, Austria). Following the recommendations of the manufacturer, extended calibration of this apparatus was done before measurements with dry air and redistilled water. Importantly, viscosity-related errors were automatically corrected in full range, which was checked using the oil N100 at 293.15 and 323.15 K. Measurements were performed with a step of 5 K and at least two independent values were obtained at each temperature. Standard uncertainties of ρ and T are $u(\rho) = 0.002 \cdot \rho$ and $u(T) = 0.01$ K, respectively.

1.2. Refractometry

The temperature-dependent refractometric studies were carried out by means of the Mettler Toledo refractometer RM40 equipped with a Peltier thermostat, which allows controlling the temperature with precision better than 0.1 K. with a resolution of 0.0001. Each alcohol was measured in the liquid phase in the temperature range of 293 – 353 K with a resolution of 0.0001.

1.3. Differential Scanning Calorimetry (DSC)

The studied collection of sterically hindered alcohols was measured calorimetrically with the use of a Mettler-Toledo DSC apparatus equipped with a HSS8 ceramic sensor (heat flux sensor with 120 thermocouples) and a liquid nitrogen cooling accessory. The temperature-dependent measurements were conducted on the samples previously poured into a sealed aluminum pan of 40 μl volume. The thermograms were collected on cooling and heating in the temperature range of 130 - 298 K in the case of 3-methyl-2-butanol and 1-cyclopropylethanol and 140-298 K in the case of the other alcohols. The cooling and heating rates were 30 and 10 K/min, respectively. The calorimetric measurements were carried out in the atmosphere of nitrogen with a flow of 60 mL/min. Glass transition temperature of each compound was determined from the heating scans as an onset of the proper thermal effect. Similar procedure was adopted for the estimation of the cold crystallization and melting points.

1.4. Broadband dielectric spectroscopy (BDS)

The dielectric studies were performed by means of a Novocontrol BDS spectrometer equipped with an Alpha Impedance Analyzer and a Quatro Cryosystem. The capacitor used for the dielectric measurements consisted of two parallel plates of 10 mm diameter made of stainless

steel, distanced with two glass fibres of 100 μm thickness and sealed with a Teflon ring. The dielectric spectra were collected in the frequency range of 10^{-1} -10^{6} Hz at a quasi-static conditions, that is after stabilization of the temperature for 3 minutes prior to each measurements using nitrogen gas with a precision better than 0.2 K. The temperature-dependent measurements were performed with a step of ΔT = 5K below glass transition or ΔT = 2K in the vicinity and above T_g.

1.5. Fourier transform infrared spectroscopy (FTIR)

FTIR measurements were carried out using the Thermo Scientific IS50 spectrometer equipped with a standard source and DTGS Peltier-cooled detector. A small amount of alcohol was put between CaF_2 glasses with 1 μm distances to preserve sample thickness. The spectra were measured in absorbance mode in the 400 – 4000 cm^{-1} range. The temperature measurements were performed during the cooling using Linkam heating/cooling stage in the broad temperature range 293 K - 113 K and the temperature stabilization 1 K/min. The spectra were recorded by the accumulation of 16 scans with a spectral resolution of 4 cm^{-1}. The obtained data in post-processing mode were subjected to the baseline, water, and carbon dioxide correction.

1.6. X-ray diffraction (XRD)

X-ray diffraction measurements were performed on a Rigaku Denki D/Max Rapid II diffractometer equipped with a rotating Ag anode, a graphite (002) monochromator and an imaging plate detector in the Debye-Scherrer geometry. The wavelength of the incident beam, λ, was 0.56 Å. The samples were measured in the liquid state, in borosilicate glass capillaries, at temperatures from the range 173 – 293 K. The temperature was controlled using Oxford Plus Compact Cooler. After subtraction of the background from the empty capillary, the diffraction intensity measured as the function of the scattering angle, 2θ, was transferred to the function of the scattering vector $Q = \frac{4\pi sin\theta}{\lambda}$. In order to compare the diffraction data for the different alcohols, the intensity functions were corrected for background, polarization, absorption, incoherent Compton scattering, and normalized to the electron units. After the data correction, the structure factor $S(Q)$ was computed as:

$$S(Q) = \frac{I(Q) - (< f^2 > - < f >^2)}{< f >^2}$$

where: $I(Q)$ is the coherently scattered intensity, normalized to electron units, $< f^2 >= \sum_{i=1}^{n} c_i f_i^2$, $< f >= \sum_{i=1}^{n} c_i f_i$, c_i and f_i are the concentration and the atomic scattering factor of the i-th atomic species, respectively, and n is the number of atomic species in the sample.

2. Supplementary information for experimental data analysis

2.1. Thermal evolution of density and refractive index

As shown in Figure S1 and Figure S2, the temperature dependences of density, *d*, and refranctive index, *n*, have a linear character for each alcohol. Therefore, the experimental data were refined with a linear function, the extrapolation of which allowed to estimate their values at lower temperature range. Noteworthy is that 3-methyl-2-butanol and 1-cyclopropylethanol are differentiated in terms of density and refractive index, despite similar molar mass. Such a feature indicates that cyclization of the isopropyl substituent exerts a huge effect on molecular packing in liquid phase. Change in the steric hindrance type from a nonaromatic cyclohexyl to the aromatic phenyl ring have also a significant impact on the refractive index values.

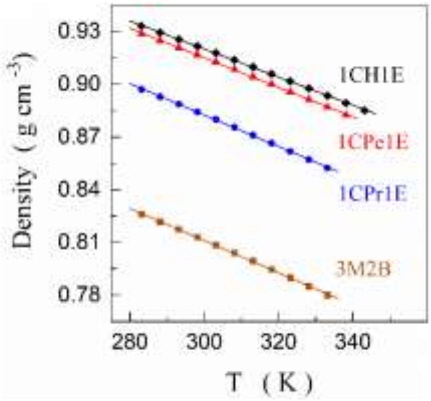

Figure S1. Thermal evolution of density of the studied alcohols.

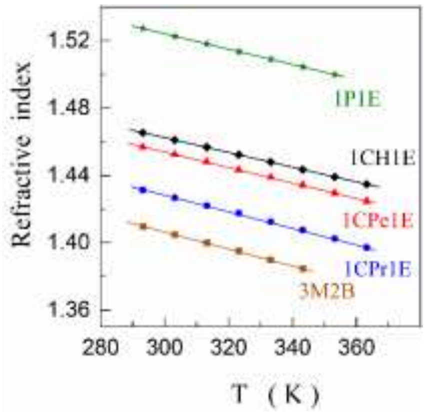

Figure S2. Temperature dependence of refractive index for each alcohol under investigation.

2.2. Dielectric data analysis

Similarly to 1-cyclohexylethanol, a single secondary relaxation occurs for the other analyzed alcohols except for 1-phenylethanol. As shown in Figure S3, maxima of this process remain visible in the frequency range of 10^{-1} -10^{6} Hz below T_g and shifts towards higher frequencies while heating.

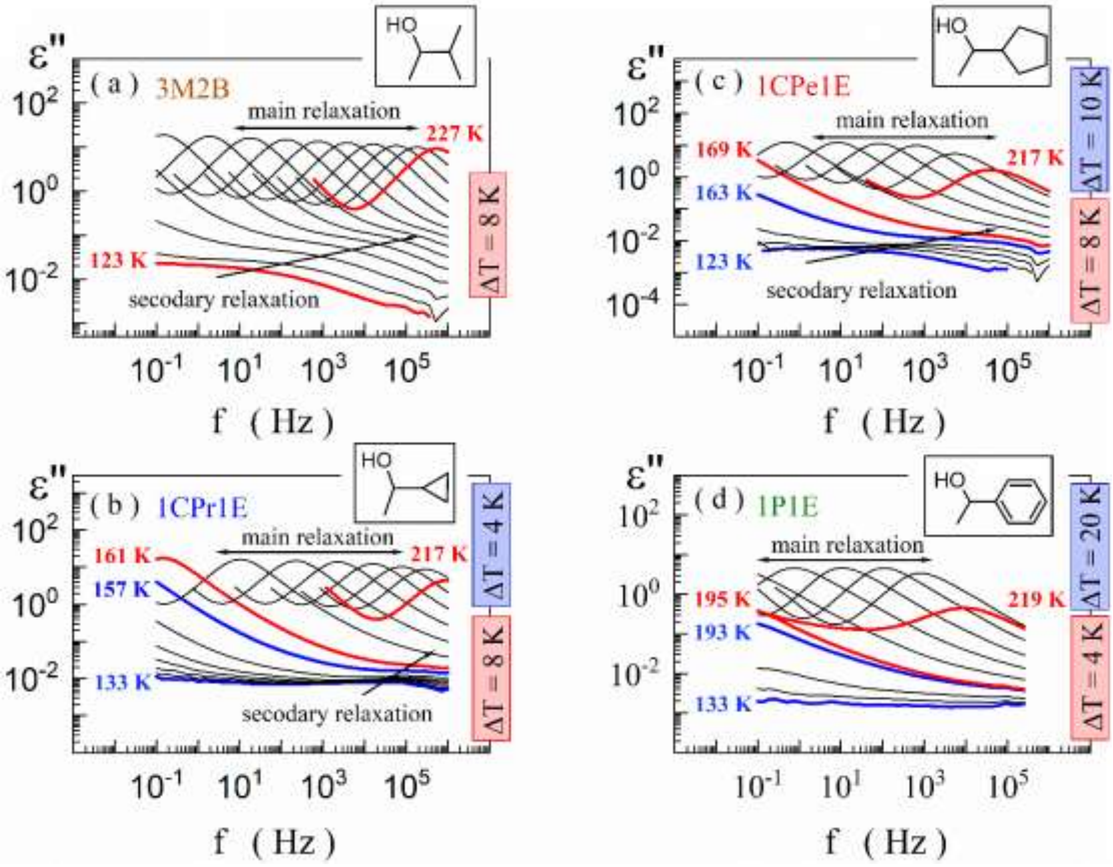

Figure S3. Dielectric loss spectra of 3-methyl-2-butanol (a), 1-cyclopropylethanol (b), 1-cyclopentylethanol (c) and 1-phenylethanol (d).

In general, secondary relaxations can be of intra- or intermolecular origin, connected with conformational changes within the molecules or limited reorientation of whole molecules, respectively. These processes which have intermolecular nature are also named as Johari-Goldstein (JG) type. In order to determine their origin, the Coupling Model (CM) or the empirical formula $E_a = 24RT_g$ should be applied. The latter approach, linking the activation energy of the secondary relaxation with the glass transition temperature, is fulfilled for the processes of intermolecular JG character. According to the CM, there is a connection between the relaxation time of a JG process (τ_{JG}), the α-relaxation time and the shape of the α-process, which can be described by the formula: $\tau_{JG}(T) \cong \tau_0(T) = t_c^{1-\beta_{KWW}} \tau_\alpha(T)^{\beta_{KWW}}$, where τ_0 is „primitive" relaxation time, t_c -crossover time from independent relaxation to cooperative

relaxation (2 ps), β_{KWW}- exponent of Kohlrausch-Williams-Watts function describing its shape. Unfortunately, due to overlapping secondary, structural and Debye relaxation processes, we were not able to determine the shape of the α-relaxation and the activation energy of the secondary relaxation in the studied compounds. Therefore, the origin of the secondary relaxation process of the studied nonaromatic alcohols remains unknown.

Due to the self-organization in chain-like associates, all compounds are characterized by occurrence of a characteristic Debye process. However, they are differentiated in terms of its amplitude. In general, the magnitude of the Debye process declines with the increasing size of the steric hindrance, which is also reflected in the decreasing static values of real part of dielectric permittivity, ε' (Figure S4).

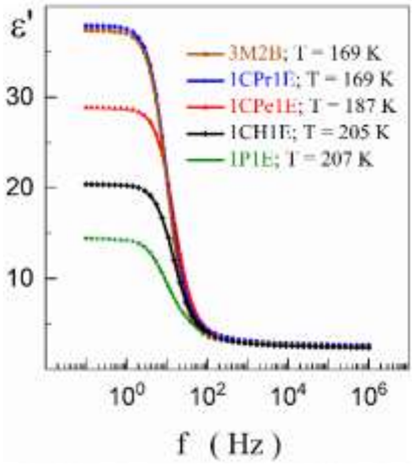

Figure S4. Comparison of the representative $\varepsilon'(f)$ spectra of the studied alcohols.

This feature indicates that the size of the H-bonded aggregates declines as the OH groups become more sterically hindered.

2.3. FTIR data analysis

Figure S5 shows the FTIR spectra of the analyzed alcohols in the high wavenumber 3650 - 3050 cm^{-1} spectral region, connected with stretching ν(OH) modes. The most prominent feature of this range is a broad γ/δ OH stretching band, which occurs for each compound. Only 1-phenylethanol is characterized by an additional blue-shifted α/β OH stretching band of lower intensity, associated with OH groups which are not involved in self-organization of molecules via O-H···O scheme.

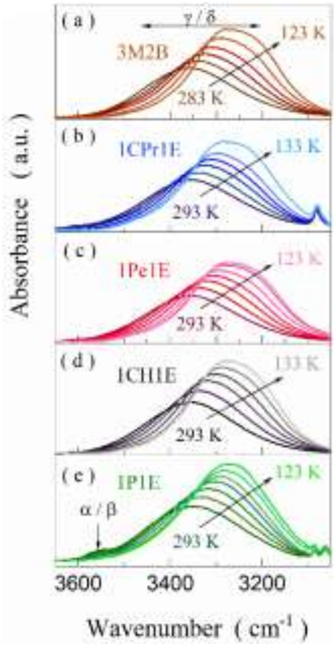

Figure S5. FTIR spectra of 3-methyl-2-butanol (a), 1-cyclopropylethanol (b), 1-cyclopentylethanol (c), 1-cyclohexylethanol (d) and 1-phenylethanol (e) in the range of 3650 - 3050 cm^{-1}.

The *γ*/*δ* OH stretching band shifts towards lower wavenumbers while cooling. Noteworthy is that its position is similar for all compounds at each isothermal conditions. However, the compounds are differentiated in terms of glass transition temperature and molecular dynamics in the liquid phase. It means, that their temperature-dependent behavior is strongly affected by the weak intermolecular interactions, such as van der Waals, π···π and O-H···π. As a consequence, in the vicinity of T_g the *γ*/*δ* OH band of 3-methyl-2-butanol and 1-phenylethanol is red- and blue-shifted the most, respectively (Figure S6). Hence, 3-methyl-2-butanol is characterized by strongest, whereas 1-phenylethanol- by the weakest H-bonds within the self-assemblies at this conditions.

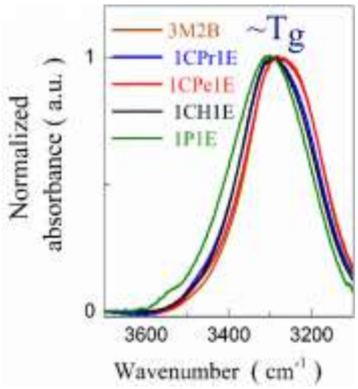

Figure S6. Comparison of the *γ*/*δ* OH band position in the vicinity of T_g.

2.4. XRD data as a function of temperature

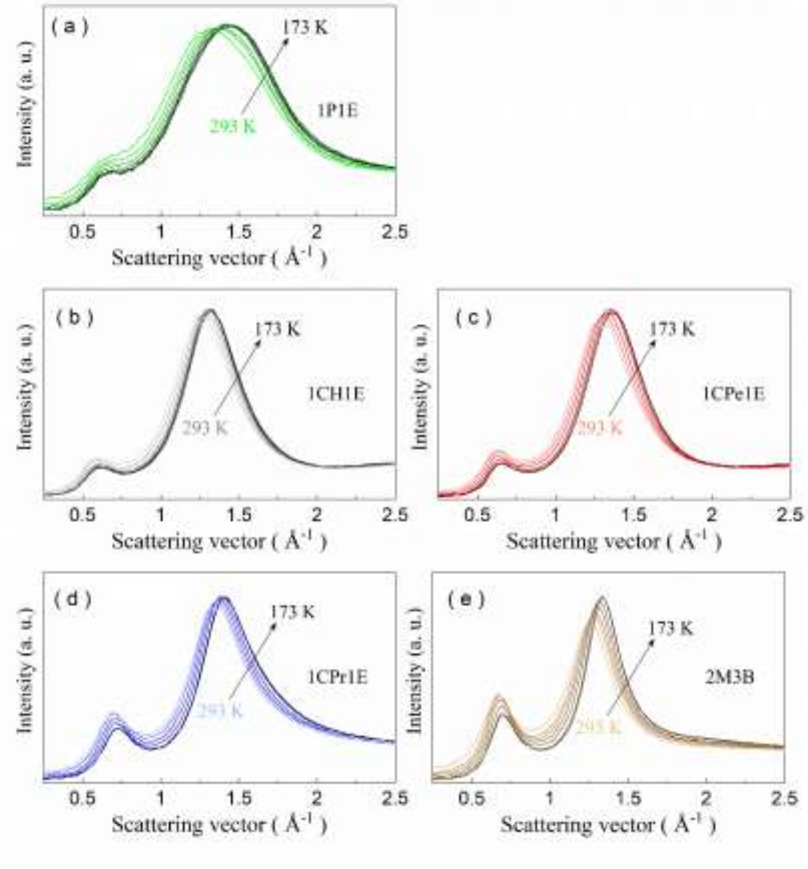

Figure S7. Temperature evolution of the diffraction patterns for the analyzed alcohols measured on cooling from 293 K down to 173 K.

Figure S7 shows the XRD patterns of the studied alcohols measured as a function of temperature. For all alcohols, common temperature dependency can be observed. The peaks shift toward greater scattering vectors with lowering the temperature. The width of the peaks decreases with lowering the temperature. The intensity of the pre-peak decreases and the intensity of the main peak increases with lowering the temperature.
